# 3D Electrospinning of Macroscopic PLLA Structures

**DOI:** 10.1002/marc.202500130

**Published:** 2025-05-12

**Authors:** Yvonne Tusiimire, Michael Lubwama, Robert Tamale Ssekitoleko, Vasileios Koutsos, Wiwat Nuansing, Norbert Radacsi

**Affiliations:** ^1^ College of Engineering, Design Art and Technology Makerere University P.O Box 7062 Kampala Uganda; ^2^ Department of Polymer Textiles and Industrial Engineering Faculty of Engineering and Technology Busitema University P.O. Box 236 Tororo Uganda; ^3^ School of Engineering Institute for Materials and Processes The University of Edinburgh King's Buildings Edinburgh EH9 3FB UK; ^4^ Department of Physiology School of Biomedical Sciences College of Health Sciences Makerere University P.O Box 7062 Kampala Uganda; ^5^ School of Physics Institute of Science Suranaree University of Technology Nakhon Ratchasima 30000 Thailand; ^6^ Center of Excellence on Advanced Functional Materials (CoE‐AFM) Suranaree University of Technology Nakhon Ratchasima 30000 Thailand; ^7^ School of Engineering Institute for Bioengineering The University of Edinburgh Mayfield Road Edinburgh EH9 3JL UK; ^8^ Centre for Cardiovascular Science The Queen's Medical Research Institute (QMRI) University of Edinburgh BioQuarter 47 Little France Crescent Edinburgh EH16 4TJ UK

**Keywords:** 3D electrospinning, 3D fibrous macrostructure, definitive Screening Design, poly L‐lactic acid, tissue engineering

## Abstract

Electrospinning typically produces 2D fibrous nanofibers with poor heavy metal sorption capabilities and weak mechanical strength without post‐processing treatment. Comparably, 3D structures have 99.992% porosity, larger pore sizes, and lower fiber density. In this study, macroscopic 3D poly L‐lactic acid (PLLA) structures are fabricated successfully by 3D electrospinning. It is found that the electrospinning solvent system, polymer concentration, phosphoric acid (H_3_PO_4_) additive concentration, collector potential, working distance, flow rate, and applied nozzle voltage affected the fiber diameter and 3D structural dimensions. The effects of these variables are investigated, and optimum conditions are obtained. The optimal parameters for the 3D PLLA structure are 0.5 wt.% phosphoric acid additive to the 12 mg mL h^−1^ PLLA solution, +1 V charged collector, +18 kV nozzle voltage, 4 cm working distance, 4 mL h^−1^ flow rate, and Dichloromethane (DCM)/ N, N‐dimethylformamide (DMF) (6:1) solvent. The structure has a 774 nm average diameter and 2.36 cm height. Scanning electron microscopy showed fiber uniformity at the different sections of the macroscopic 3D PLLA structures. These results expand the possibilities of using PLLA as 3D electrospun biomimetic structures.

## Graphical abstract

This study uses a modified 3D printer to fabricate macroscopic electrospun 3D PLLA nanofibrous structures. It discusses the effects of process, solution, and ambient parameters. The optimum set of variables is zrealized with a uniform nanofibrous distribution in the bottom, middle, and top structural layers. These structures have fewer limitations compared to 2D electrospun flat mats and are desirable for biomedical and energy applications, filtration, and insulation.

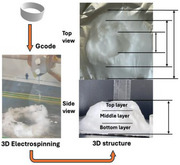

## 1 Introduction

Electrospinning (ES) is a quick, scalable, and inexpensive method of assembling nanofibers, making it a popular nanofabrication technique already used for industrial‐scale production. The structural characteristics include a high fiber diameter‐to‐length ratio and high porosity from various monomeric^[^ ^1^ ^]^ or polymeric‐based materials,^[^ ^2^ ^]^ ideal for technical uses like medical, filtration, apparel, and geotextiles.^[^ ^3^, ^4^ ^]^ Typically, ES produces 2D fibrous mats or 3D, which is desirable for its application in biomedical, extra‐cellular matrix mimicking,^[^ ^5^ ^]^ energy, catalysis, filtration, food industry, cosmetics, heat,^[^ ^6^ ^]^ and sound insulation.^[^ ^7^, ^8^ ^]^ 3D nanofibrous structures address 2D structural limitations like poor sorption capabilities for heavy metals^[^ ^9^ ^]^ and weak mechanical strength without post‐processing treatment.^[^ ^10^ ^]^ Compared to 2D structures, 3D structures are more porous,^[^ ^11^have larger pore sizes and lower fiber density.^[^ ^12^ ^]^

**Figure 1:**
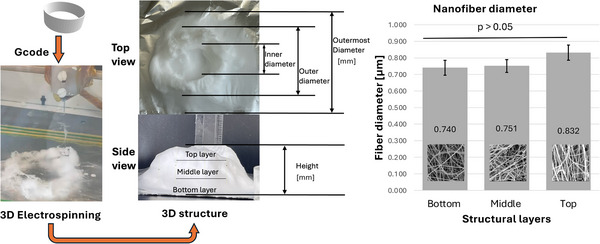
Overview of the 3D electrospinning structural formation and analysis.

A simple method for producing macroscopic sponge‐like 3D nanofibrous mats is by 3D electrospinning (3D ES).^[^ ^13^ ^]^ Compared to wet,^[^ ^14^, ^15^ ^]^ template‐assisted,^[^ ^16^ ^]^ multilayering,^[^ ^17^ ^]^ cold‐plate,^[^ ^18^ ^]^ and freeze‐drying‐casting^[^ ^19^, ^20^ ^]^ ES methods, which require time‐consuming additional steps, 3D ES is based on self‐assembly, meaning that it can be done in one step without any modifications to the ES setup.^[^ ^21^ ^]^ Introducing a combination of 3D printing and 2D electrospinning provided a platform for quickly producing 3D ES. In this method, the nozzle movement is guided by the computer‐aided design controlling the *x–y* axis^[^ ^22^, ^23^ ^]^.

Multiple factors are monitored to achieve the desirable nanofibers and are categorized as process, solution, and ambient parameters.^[^ ^21^, ^24^, ^25^ ^]^ These factors affect the fiber quality and the overall structure.^[^ ^26^ ^]^ For any successful ES, optimizing is critical^[^ ^27^, ^28^ ^]^ as it introduces factor calibration complexity for desirable output. The common optimizing technique is a one‐factor‐at‐a‐time analysis where precise factor levels are deducted while keeping the rest constant. This technique disregards factor combinations as it assumes each factor is at the same level. Given that ES is a multifactor method, using this optimizing technique will only explore partial factor‐level interactions.

Poly L‐lactic acid (PLLA) is an FDA‐approved biocompatible and biodegradable polymer used in pharmaceutics and medical medicine for drug delivery systems, sutures, scaffolds, films, and implants.^[^ ^29^ ^]^ PLLA is also a promising degradable piezoelectric material. Therefore, a macroscopic sponge‐like 3D structure would likely enhance PLLA's potential for piezoelectric energy generation.^[^ ^13^ ^]^

Electrospinning is a multi‐factorial process requiring optimization to acquire a desirable structure. This study investigates the effects of 3D ES factors on both the macroscopic and microscopic morphology using PLLA, utilizing a Definitive Screening Design (DSD). These factors include polymer concentration, solvents, phosphoric acid (H_3_PO_4_) additive, flow rate, nozzle voltage, collector potential, and nozzle speed. To acquire a distinct 3D shape, the nozzle followed a designed pattern. This study used a 55 mm hollow cylinder pattern that was designed and sliced to generate a G‐code that was fed into a modified 3D electrospinning apparatus. The influence of 3D ES factors on the 3D structural dimensions and nanofiber diameters was measured and analyzed. **Figure** **1** shows the overview of this process.

## 2 Results and Discussion

### 2.1 Definitive Screening Design

DSD analysis identifies influential factors (Sparsity of Effects) by analyzing linear, nonlinear, and quadratic effects,^[^ ^30^ ^]^ thus in‐depth interactions.^[^ ^31^ ^]^ It obtains experimental runs by assuming that there are only a couple of key factors that affect the actual response surface identified from the full quadratic model (first and second order) ^[^ ^30^ ^]^ For this reason, using Minitab (v 19 2020 2.0), eight ES factors were investigated, as shown in **Table** **1**. These factors were adopted by Vong et al.,^[^ ^23^ ^]^ and Chinnakorn et al.,^[^ ^32^ ^]^ and they included working distance (between 2–6 cm), flow rate (between 0.2–0.6 mL h^−1^), nozzle speed (between 1–12 mm s^−1^), nozzle voltage (between 16–20 kV), collector potential (negative, grounded or positive), polymer concentration (9–12 mg mL^−1^), phosphoric acid (H_3_PO_4_) additive concentration (0–1 wt.%), and the solvents Dichloromethane (DCM)/ N, N‐dimethylformamide (DMF) (6:1) and Trifluoroacetic acid (TFA).

**Table 1:** Definitive Screening Design showing factors considered.

Factors	Levels		
Categorical Factor	1	2	
Solvent	DCM/DMF	TFA	
Continuous Factors	**−1**	0	1
Polymer conc. (mg mL^−1^)	9	12	15
Additive conc. (wt.%)	0	0.5	1
Collector potential (V)	−1	0	1
Working Distance (cm)	2	4	6
Flow rate (mL h^−1^)	2	4	6
Nozzle Speed (mm s^−1^)	1	6.5	12
Nozzle voltage (kV)	16	18	20

Furthermore, two responses were considered: average fiber diameter extracted from the bottom layer and macroscopic height. DSD analysis showed model *p*‐values of 0.001 and 0.052 for fiber diameter and height, respectively, as shown in **Table** **2**. Considering 95% confidence, the average fiber diameter DSD model is statistically significant. Furthermore, the model identified solvent (p = 0.001) for further investigation. On the other hand, the 3D height response was statistically insignificant, and no influential factor was identified as significant. Identifying one factor, solvent, renders DSD ineffective for this study.

**Table 2:** Summary of Definitive Screening Design Analysis showing Pareto charts and identified factors using two responses: average fiber diameter bottom layer and average height of *p* < 0.05 significance.

	Average fiber diameter bottom layer	Average height
Pareto Charts	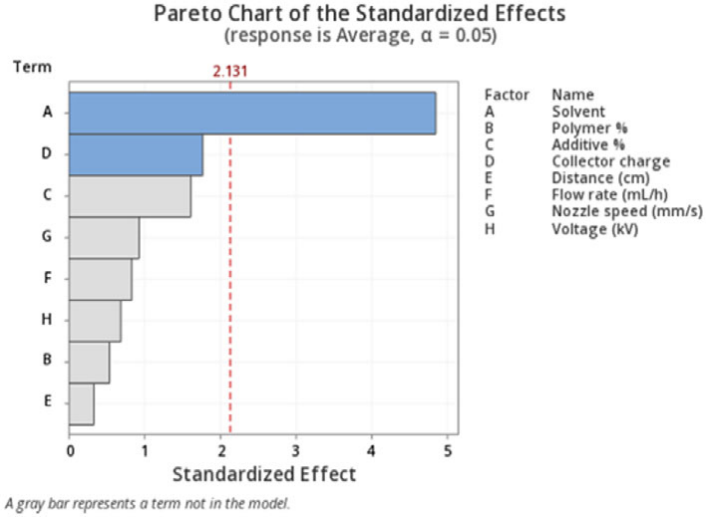	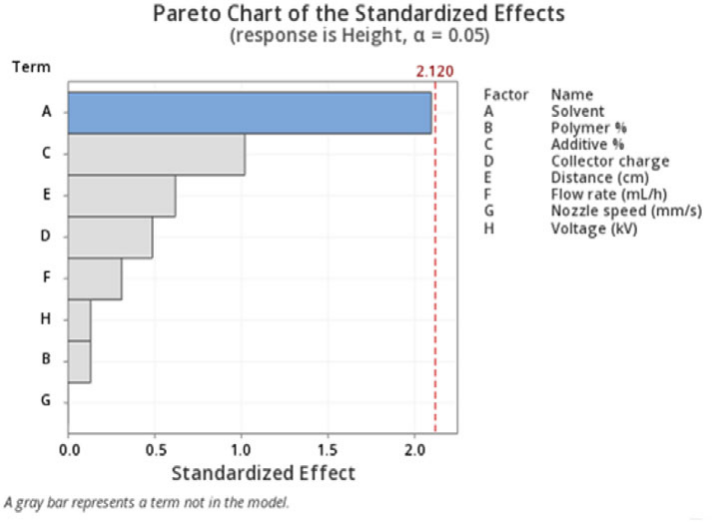
Model *p*‐value	0.001	0.052
Identified factor	Solvent	None
Factor *p*‐value	0.001	N/A

DSD's choice of one relevant factor instead of multiple factors defies the purpose. This choice could have been influenced by other factors that were kept constant, like nozzle diameter,^[^ ^28^ ^]^ ambient range,^[^ ^1^, ^33^ ^]^ collector type^[^ ^4^ ^]^ working distance, additive type, and experimental time,^[^ ^4^, ^25^, ^27^, ^34^ ^]^ despite careful selection from a pool of multiple factors.^[^ ^21^, ^35^ ^]^ Therefore, to decisively eliminate levels with a given factor, the factor interaction was based on observations during experimental runs, as shown in **Table** **3**. Observations of each factor are hence discussed.

**Table 3:** Summary of observations of the Definitive Screening experiment runs (DSD 1–18) derived from polymer concentration, additive concentration, collector potential, working distance, flow rate, nozzle speed, and nozzle voltage against two responses: average fiber diameter and average height.

Run Order	Solvent	Polymer conc. (mg mL^−1^)	Additive conc. (wt.%)	Collector potential (V)	Distance (cm)	Flow rate (mm s^−1^)	Nozzle Speed (mm s^−1^)	Nozzle voltage (kV)	Average Fiber (µm)	Height (mm)	Observations
DSD 1	TFA	15	1	0	2	6	1	16	0	0	Electrospray
DSD 2	DCM/DMF	15	0.5	1	2	2	1	20	0.84	0	Clog, Current discharge
DSD 3	DCM/DMF	15	1	−1	4	2	12	16	2.66	3	Bridge, Jets
DSD 4	DCM/DMF	12	0.5	0	4	4	6.5	18	0.65	25	Drip, Stand
DSD 5	DCM/DMF	12	0	1	2	6	12	16	1.60	0	Bridge, Jet, Electrospray
DSD 6	DCM/DMF	9	1	−1	2	6	6.5	20	1.80	3	Bridge, Electrospray, Stand
DSD 7	TFA	9	0	1	4	6	1	20	0	0	Electrospray, Recede
DSD 8	TFA	15	0	−1	2	4	12	20	0	0	Electrospray
DSD 9	TFA	12	1	−1	6	2	1	20	0	0	Single fiber stand, Electrospray
DSD 10	TFA	9	1	1	2	2	12	18	0	0	Stand, Current discharge
DSD 11	TFA	12	0.5	0	4	4	6.5	18	0	0	Electrospray
DSD 12	TFA	15	0	1	6	2	6.5	16	0	0	Recede, Electrospray
DSD 13	TFA	9	0.5	−1	6	6	12	16	0	0	Electrospray
DSD 14	DCM/DMF	9	1	1	6	4	1	16	1.24	10	Blockage, Bridge jets stand
DSD 15	DCM/DMF	15	0	−1	6	6	1	18	1.49	0	Clog, Jets
DSD 16	TFA	9	0	−1	2	2	1	16	0	0	Recede, Solvent
DSD 17	DCM/DMF	9	0	0	6	2	12	20	1.04	0	Stand
DSD 18	DCM/DMF	15	1	1	6	6	12	20	1.8	13	Bridge, Structure rose

#### 2.1.1 Effect of Solvents

The effect of the electrospinning solvent on 3D structure was investigated using two different solvent systems: a mixture of DCM/DMF (6:1) and pure TFA. Results in Table 3 show bottom layer fiber diameter averages ranged from 0.65 to 2.66 µm for DSD 4 and DSD 3, respectively, while structure height ranged from 3 to 25 mm for DSD 3 and DSD 4, respectively. DCM/DMF solutions mixtures were successfully applied to electrospin 3D PLLA, while pure TFA solution failed to produce nanofibers. The failure to electrospun PLLA with TFA in our study contradicts previous studies,^[^ ^36^, ^37^ ^]^ even with improved conductivity.^[^ ^38^ ^]^ Increased conductivity with H_3_PO_4_ additive concentrations, as shown in **Table** **4**, is said to facilitate ES and 3D buildup.^[^ ^22^, ^23^, ^32^ ^]^ Increased conductivity should facilitate 3D buildup by increasing the polarizability of electrospun fibers, causing repulsive forces between fibers.^[^ ^22^, ^23^ ^]^ Due to the DCM/DMF solution's success in producing nanofibers, it was chosen for further investigations.

**Table 4:** Summary of the conductivity of polymer solutions with DCM/DMF and TFA solvents for 0, 0.5, and 1% additive concentrations.

	Conductivity (mS cm^−1^)		
H_3_PO_4_ Additive conc.	0 wt.%	0.5 wt.%	1 wt.%
DCM/DMF	1.85	2.96	3.82
TFA	2.97	3.54	4.01

#### 2.1.2 Effect of Polymer Concentration

The effect of polymer concentration was investigated at 9, 12, and 15 mg mL^−1^. The resulting PLLA solutions at these concentrations were runny, smooth, and viscous, respectively. The runny solution at 9 mg mL^−1^ (DSD 6) enabled electrospraying, which was attributed to the low polymer concentrations. Low polymer concentrations lead to weak intermolecular interactions, resulting in low viscosity and causing jet break‐ups to transform into droplets before reaching the collector.^[^ ^29^ ^]^ An increase in concentration led to the formation of beads, as shown by DSD 6 and DSD 14 in **Table** **5**, while further increases converted these beads into smooth fibers. Smooth fibers form at the critical polymer concentration, facilitating a steady solution flow. Beyond this concentration, viscosity increases, hindering solution flow and leading to clogging.^[^ ^39^ ^]^ Therefore, the critical value was determined to be 12 mg mL^−1^ PLLA, which was chosen for further investigation.

**Table 5:** Summary of fiber diameters (µm) obtained from SEM images of DSD runs with a scale bar of 10 µm. DSD runs varied polymer concentrations, additive concentrations, collector potential, working distance, flow rate, nozzle speed, and nozzle voltage.

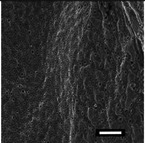	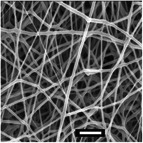	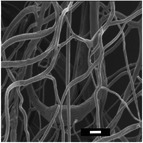	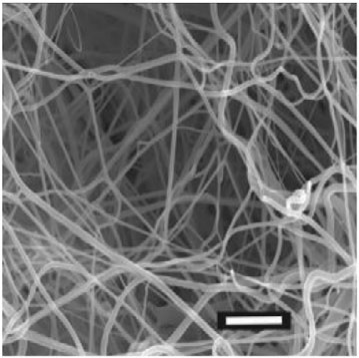	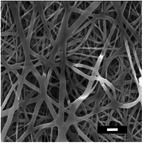
DSD 10.00 µm	DSD 20.84 ± 0.18 µm	DSD 32.70 ± 1.76 µm	DSD 40.65 ± 0.28 µm	DSD 51.60 ± 0.57 µm
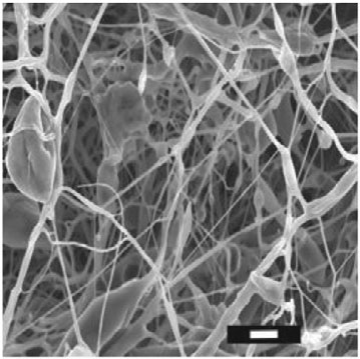	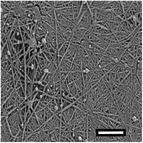	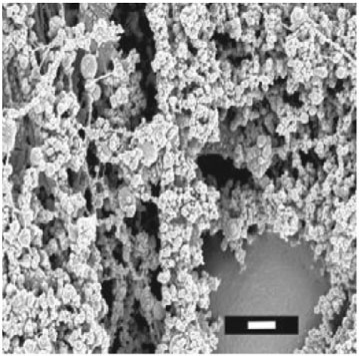	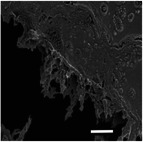	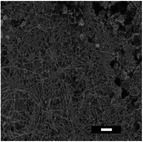
DSD 6 1.80 ± 1.40 µm	DSD 7 0.00 µm	DSD 10 0.00 µm	DSD 11 0.00 µm	DSD 12 0.00 µm
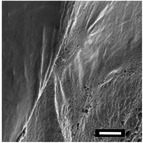	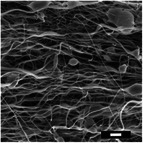	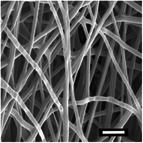	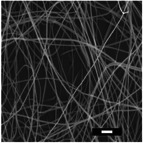	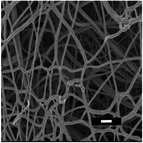
DSD 13 0.00 µm	DSD 14 1.24 ± 2.00 µm	DSD 15 1.50 ± 0.28 µm	DSD 17 1.04 ± 0.17 µm	DSD 18 1.80 ± 0.62 µm

#### 2.1.3 Effect of Working Distance

The effect of the working distance on the 3D structure was investigated using 2, 4, and 6 cm distances. When combined with the electrostatic potential, the working distance between the two electrodes determines the strength of the field and the total time it takes for a fiber to reach the collector.^[^ ^27^, ^40^ ^]^ Two contradictory theories explain the working distance on the fiber diameter. One theory states that an increase in the working distance results in a smaller fiber diameter due to an increase in the evaporation time, an increase in the time for instability of the bending/folding interval, and thus greater drafting.^[^ ^41^ ^]^ This could explain the 1.8µ m diameter at a 2 cm distance with a 20 kV potential difference (DSD 6). The alternative theory suggests that thick fibers are formed at larger distances instead due to a weakened electrostatic field, resulting in reduced drafting.^[^ ^2^ ^]^ This could explain the 2.66 µm diameter at 6 cm with 16 kV (DSD 3).

Furthermore, at 2 cm, strong electric fields became intense enough to cause a current discharge, which subsequently terminated the experiment. At 6 cm, the electrodes were too far apart to enable 3D buildup, supporting Chinnakorn et al.,^[^ ^32^ ^]^ who stated that 3D ES lies between melt electro‐writing and conventional ES, both of which depend on distances within the straight jet and the onset of jet instability. For these reasons, 4 cm was chosen to facilitate the 3D buildup.

#### 2.1.4 Effect of Applied Voltage on the Nozzle

The voltage effect on 3D ES was investigated at +16, +18, and +20 kV potential on the nozzle while the collector potential was −1, 0, or +1 V. The positive and negative collector potential was applied using a DC power supplier, while grounded was indicated as 0 V potential. This potential was also investigated at −1 and +1 kV with no noticeable results compared to −1 and +1 V. The applied positive nozzle voltage ionizes the polymeric solution inside it, and a Taylor cone erupts when the reciprocal repulsion of charges is greater than the surface tension of the solution.^[^ ^25^, ^33^ ^]^ It has been argued that decreased voltage potential increases fiber diameter, which is attributed to reduced Coulombic force.^[^ ^42^ ^]^ This force causes insufficient charge repulsion enough to draft, further reducing solvent evaporation.^[^ ^25^ ^]^ This could explain why, at the lowest tested applied voltage (+16 kV), 2.66 µm fiber diameter was collected with the solution tending to solidify at the nozzle tip. It also explains the drafting of 0.84 µm fiber at 20 kV.^[^ ^28^ ^]^ In hindsight, experiments conducted at +20 kV with working distances of 2 or 4 cm tended to current discharge, and evidence of the corona effect was observed.^[^ ^27^ ^]^ Consequently, +18 kV was selected as the applied voltage on the nozzle.

#### 2.1.5 Effects of Flow Rate

Flow rate effects on the 3D ES were investigated using 2, 4, and 6 mL h^−1^. Ideally, the flow rate critical value is when the dispersed solution immediately transforms into dry fibers at the collector with adequate solvent evaporation. When flow is below the critical value, receded jets, as seen at 2 mL h^−1^, occur.^[^ ^28^, ^40^ ^]^ When above the critical value, the dispersed solution is not taken up, causing solidification at the nozzle tip or bridging, dripping, and multiple jets^[^ ^40^ ^]^ observed with 6 mL h^−1^. For these reasons, 4 mL h^−1^ was selected for proceeding investigations.

#### 2.1.6 Effect of Nozzle Speed

This investigation considered 1, 6.5, and 12 mm s^−1^. This speed affects the placement of the nanofiber as the nozzle follows the coded pattern. At 1 mm s^−1^, the 3D ES structure was more compact compared to increased speeds, 6.5 and 12‐mm s^−1^, which created fluffy structures. Low speeds also improve precise fiber deposition, adequately building a 3D structure.^[^ ^32^ ^]^ For these reasons, 1 mm s^−1^ was selected.

### 2.2 Two Factor‐3 Levels Design

Constant and varied factors were considered to study 3D ES PLLA further. As described above, the constant factors were established by decisively eliminating levels from DSD observations. Consequently, the constant factors included a 12 mg mL^−1^ PLLA polymer concentration, an 18 kV applied high voltage, a 4 cm working distance, a 1 mm s^−1^ nozzle speed, a 4 mL h^−1^ flow rate, and a DCM/DMF (6:1) solvent. In contrast, the varied factors comprised 0–1 wt.% additive and collector charge settings of ground, float, and +1 V, where ground represents when a grounding cable was connected to the collector, float was when there was no grounding cable connected, and +1 V was when the cable connected had a +1 V potential. These parameters led to nine experiments, as detailed in **Table** **6**. Three responses were evaluated: 3D structural height, 3D structural diameter, and nano‐fiber diameter.

**Table 6:** Data summary showing additive concentration and collector charge showing 3D structure's top and side view, SEM of the bottom layer, fiber diameter (bottom (BF), middle (MF), and top fiber (TF), structural dimensions of outer‐most (OMD), outer diameter (OD), inner diameter (ID), and height. Photograph scale bar: 10 mm. SEM scale bar: 10 µm.

Exp	Additive conc. (wt.%)	Collector potential (V)	3D ES structure		SEM	Fiber diameter (µm)			3D Structural Dimensions (mm)			Height (mm)
			Top view	Side view	Bottom layer	BF	MF	TF	OMD	OD	ID	
0F	0.0	Float	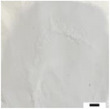	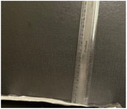	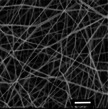	0.65	0.00	0.00	124.08	0.00	0.00	0.00
0G	0.0	Ground	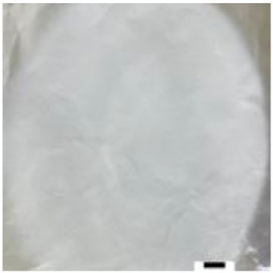	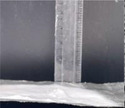	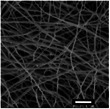	0.68	0.00	0.00	121.11	0.00	0.00	0.00
0P	0.0	+1 V	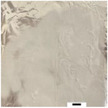	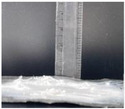	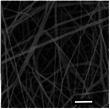	0.80	0.00	0.00	72.50	0.00	0.00	0.00
0.5F	0.5	Float	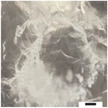	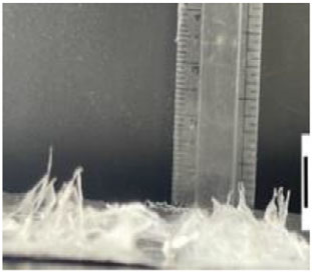	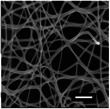	0.94	0.00	0.00	75.50	0.00	43.34	10.39
0.5G	0.5	Ground	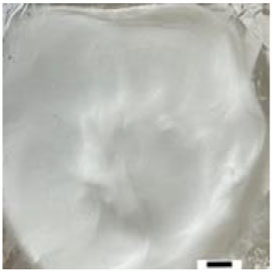	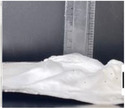	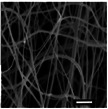	1.00	0.93	0.80	105.29	36.49	14.81	16.40
0.5P	0.5	+1 V	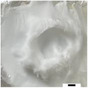	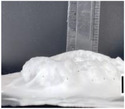	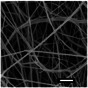	0.74	0.75	0.83	111.95	69.30	36.12	23.58
1F	1.0	Float	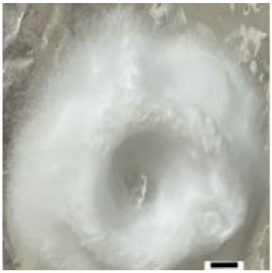	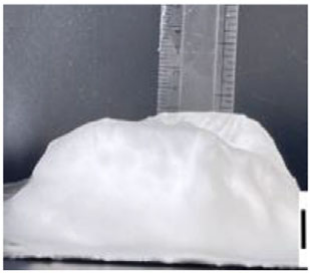	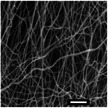	0.49	0.76	0.81	95.26	59.32	33.35	30.60
1G	1.0	Ground	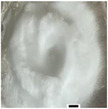	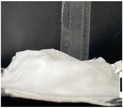	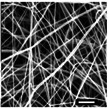	0.70	0.79	0.92	100.73	66.14	24.97	27.04
1P	1.0	+1 V	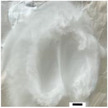	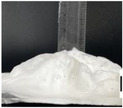	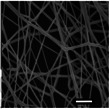	0.92	1.00	1.08	95.08	48.58	26.21	23.28

#### 2.2.1 Effect on Fiber Diameter

SEM images were analyzed to investigate the effects of collector charge and additive concentration on nanofiber diameter. ImageJ analyzed 80 fiber diameters of SEM images extracted from the bottom, middle, and top 3D ES layers, as shown in Figure 4. Larger fiber diameters were observed at 1P top fibers with 1.1 µm while the smallest at 1F bottom fibers with 0.5 µm. Larger fibers found at the top layer could be due to less evaporation time and thus shorter draft due to the shortened distance during 3D buildup compared to longer evaporation and drafting time for bottom layer fibers^[^ ^23^, ^41^ ^]^.

**Figure 2:**
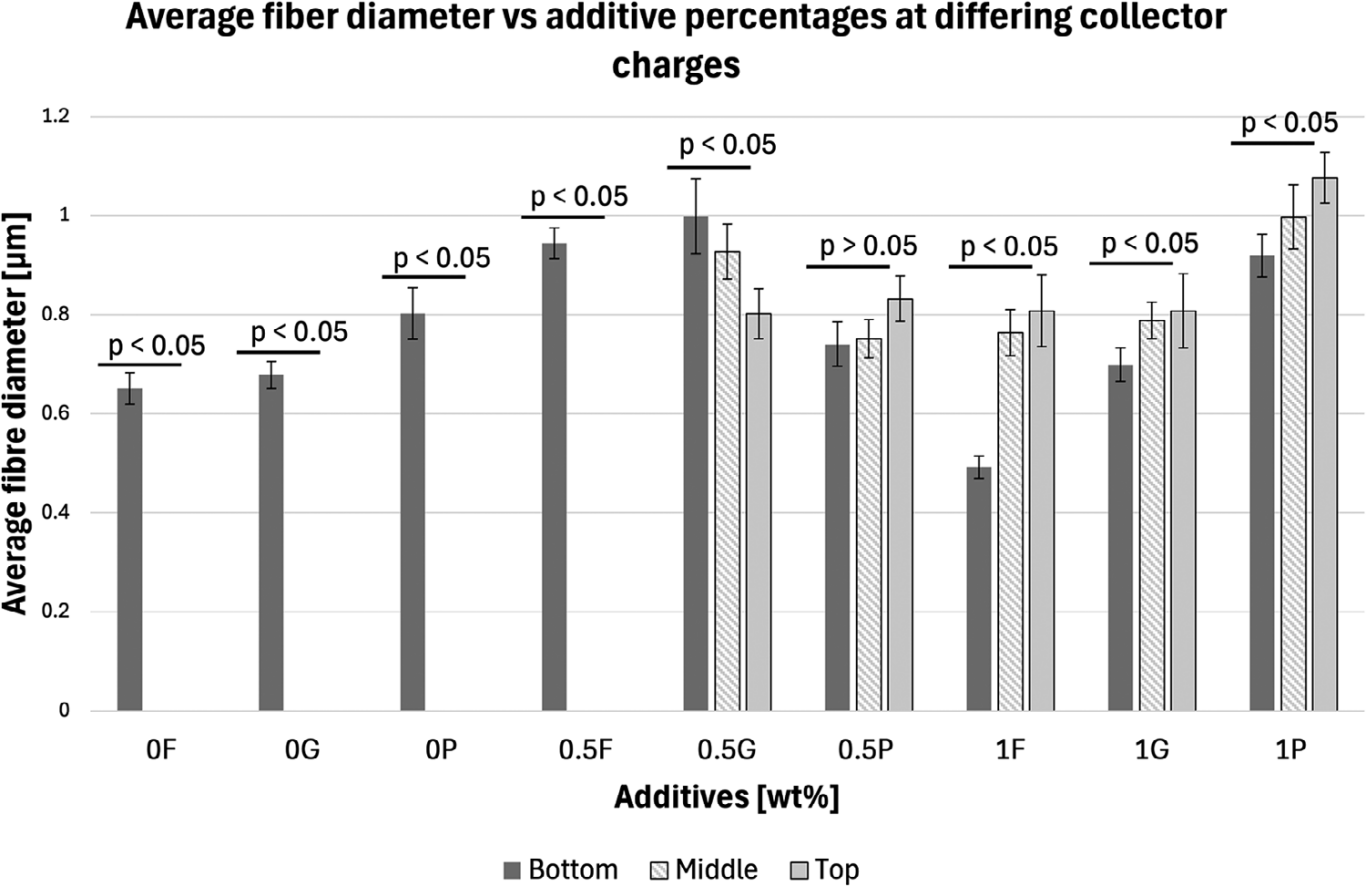
A graph showing the average fiber diameter of the bottom, middle, and top layers of *p* < 0.05 significance showing 0, 0.5, and 1 wt.% additive concentration and float (F), grounded (G), and 1 V positive (P) collector potential.

Furthermore, apart from 0.5P, other experiments had a *p*‐value greater than 0.5. At 0.5 wt.% additive, +1 V collector charge (0.5P), the *p*‐value was less than 0.05 in **Figure** **2**, showing statistical insignificance. This implies that fibers extracted from the bottom, middle, and top layers showed no significant differences and were thus homogeneous. While the other experiments showed *p*<0.05, implying statistical significance, meaning that the bottom, middle, and top fiber diameters were distinct.

**Figure 3:**
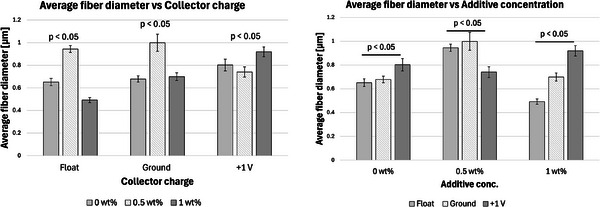
Graphs illustrating the average fiber diameter of the bottom layer compared to the collector charger (float, grounded, and 1 V positive) and height in relation to the additive conc. (0, 0.5, and 1 wt.%) with *p* < 0.05 significance.

Furthermore, considering the bottom fibers, there was a statistically significant relationship between additive concentration and fiber diameter, as shown in **Figure** **3**. No correlation was identified. For instance, 0.5 wt.% additives registered bigger diameters at floated and grounded collectors and lower diameters at a +1 V charged collector. The middle and bottom layers are shown in **Table** **7**. Also, see the supporting information.

**Table 7:** Summary of SEM images (10 µm scale bar) showing the average fiber diameter of the middle and bottom layer 3D ES nanofibers where G, P, and F are grounded, positive, and floating collector charges, respectively, and 0.5 and 1 are the additive wt.%.

		Average fiber diameter (µm)			
	0.5G	0.5P	1F	1G	1P
Middle layer	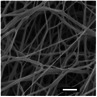 0.93 ± 0.25	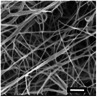 0.75± 0.17	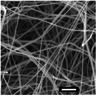 0.76 ± 0.21	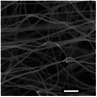 0.79 ± 0.17	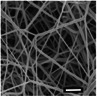 1.0± 0.29
Top layer	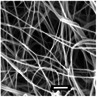 0.80± 0.23	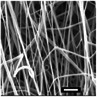 0.83 ± 0.21	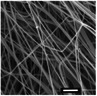 0.81 ± 0.33	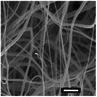 0.92 ± 0.34	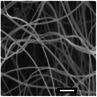 1.08± 0.23

#### 2.2.2 Effect on Height

The 3D height measurement was extracted from the side view pictures, as shown in Table 4. All experiments with no additive, 0F, 0G, and 0P, remained 2D while the rest experienced a 3D buildup. An average of 5 measurements was taken because the structures had short and tall areas. The average height ranged from the tallest, 30.6 mm, and shortest, 10.39 mm, for experiments 1F and 0.5F, respectively. Furthermore, the positive collector showed a negligible height difference of 23.6 and 23.3 mm for 0.5 and 1 wt.% additive concentration, respectively.

**Figure 4:**
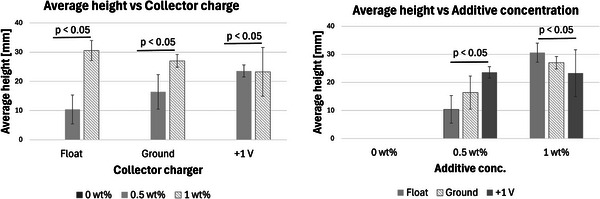
Graphs showing average height versus collector charger (float, grounded, and 1 V positive) and height versus additive concentration (0, 0.5, and 1 wt.%) of *p* < 0.05 significance.

There is a positive correlation between the average height and the additive concentrations, as shown in **Figure** **4**. The grounded collector, for instance, registered a height increase from 0 to 16.4 to 27 mm as the additive concentration increased from 0 to 0.5 to 1 wt.%, respectively. This positive correlation was also seen with the floated collector. Vong et al.^23]^ and Chinnakorn et al.^[^ ^32^ ^]^ affirmed this observation, explaining that an increase in additive wt.% increases surface charge, enabling 3D structural support and buildup. The surface charge is due to the additional free ions in the solution increasing the conductivity, as shown in Table 4. Sun et al.^[^ ^43^ ^]^ explains that due to the polarization effect, as fibers are deposited on the collector, oppositely charged fibers are attracted toward the electrospinning needle facilitating the 3D build‐up.^[^ ^43^ ^]^ Beyond the additive wt.% critical value, cotton‐like structures are produced instead^[^ ^23^, ^32^ ^]^.

On the other hand, results showed that the collector charge affected the structural height with *P* < 0.05. For instance, at 0.5% additive, the average height was 10.4, 16.4, and 23.6 mm for float (0.5F), ground (0.5G), and +1 V (0.5P) collector charge, respectively. At 1%, the tallest and shortest heights were registered at float and +1 V, respectively. Although the effects of collector charge and height are not fully understood, there is an apparent effect given the statistical significance *p* < 0.05.

#### 2.2.3 Effects on 3D Structural Dimensions

**Figure 5:**
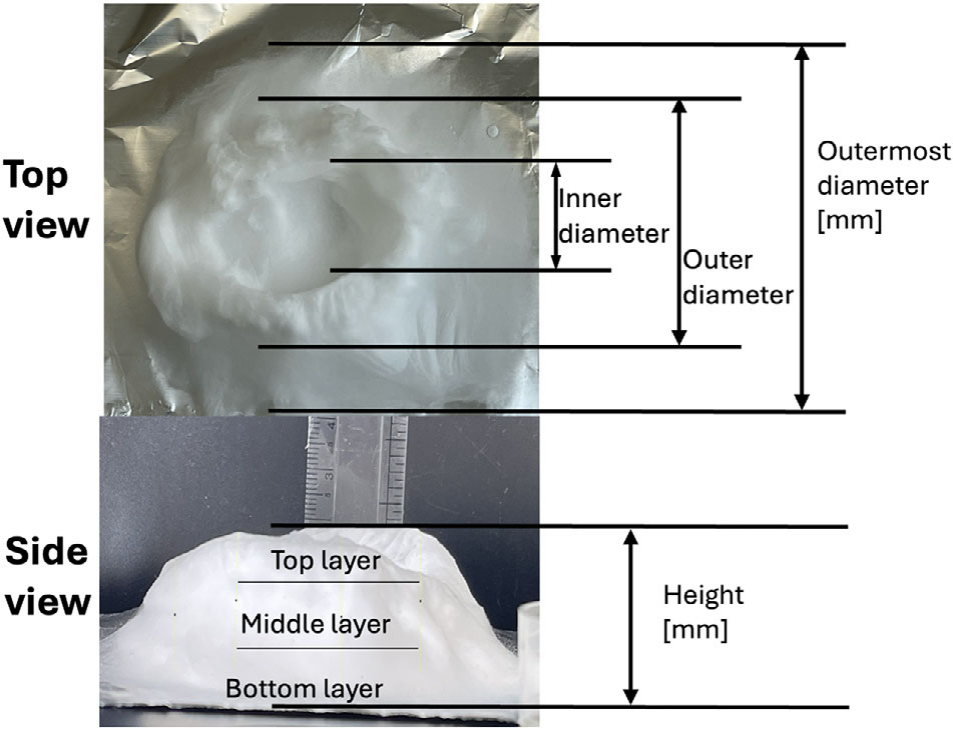
Dimensions of the top and side view of the 3D ES structure.

The 3D Structural dimensions were extracted from the top view pictures, as shown in Table 6. These diameters included the outermost (OMD), outer (OD), and inner (ID) diameters referenced in **Figure** **5**. The outermost diameter ranged from the widest at 124.08 to 72.5 mm for 0F and 0P, respectively. The outer diameter ranged from 69.3 to 36.49 mm for 0.5P and 0.5G, respectively, while the inner diameter ranged from 43.34 to 14.81 mm for 0.5F and 0.5G, respectively. 2D structures, 0F, 0G and 0P registered only the outermost diameters.

Given that the nozzle followed a 55 mm circular motion, the diversion can be explained by the whipping angle^[^ ^22^ ^]^ and whipping diameter. The whipping diameter in stationary single nozzle ES is registered as the sample diameter and correlates with working distance. As the nozzle moves in 3D ES, so does the whipping action. Thus, the moving whipping diameter expands the deposition area. This could explain how a nozzle circular movement flowing a 55 mm diameter creates a 124.08 mm structural diameter (0F). This agrees with Vong et al.,^[^ ^23^ ^]^ who state the impossibility of obtaining the exact 3D structural shape while 3D ES. Furthermore, on close inspection of 1P, a second inner diameter, which could be the whipping diameter, is clearly visible.

## 3 Conclusion

This study investigated optimizing poly‐L‐lactic acid (PLLA) 3D electrospinning parameters. To obtain the 3D ES PLLA structure, a 55 mm hollow cylinder pattern with a 21G hypodermic needle on a 5 mL syringe for 10 min running time with a stationary aluminum‐wrapped collector plate was used. Two sets of experiments were run while analyzing the statistical significance when *p* < 0.05.

The first set of experiments used the definitive screening design (DSD) method to identify influential factors out of the eight varied. The varied factors included solvent, polymer concentration, additive concentration, collector charge, working distance, flow rate, nozzle speed, and applied voltage. Although DSD identified only solvent as the singular influential factor, further analysis was explored. The study deductively eliminated factor levels based on their interactions with other factors while exploring fiber diameter and 3D structural height responses. Upon this analysis, 3D ES was possible at 18 kV applied high voltage, 4 cm working distance, 1 mm s^−1^ nozzle speed, 4 mL h^−1^ flow rate, DCM/DMF (6:1), and 12 mg mL^−1^ PLLA concentration. Furthermore, additive concentration and collector charge were identified for further analysis, creating the second set of experiments.

The second set of experiments ranged from two factors: 0–1 wt.% additive concentration range and the collector charge at ground, float, and +1 V generating. This generated 0F, 0G, 0P, 0.5F, 0.5G, 0.5P, 1F, 1G, and 1P. To understand these factors, three responses were investigated, including nano‐fiber diameter, 3D structural dimensions, and 3D height. The fiber diameter was extracted from three distinct layers: the bottom layer (BF), middle layer (MF), and top layer (TF). The structural diameter included the outermost diameter (OMD), outer diameter (OD), and inner diameter (ID). At 0 wt.% additive, 2D ES was achieved with neither a structural height nor an OD or ID, while 3D ES was possible for 0.5 and 1 wt.% additives. Although the nozzle followed a 55 mm circular pattern, the OMD ranged from the widest at 124.08 mm and the narrowest at 72.5 mm for 0F and 0P, respectively. Furthermore, there was a positive correlation between additive concentration and structural height, with the highest being 30.6 mm at 1F and the shortest being 10.39 mm at 0.5F. Although 1F had the lowest average diameter at 0.49 µm (bottom fibers) and the tallest 3D at 30.6 mm, the average fibers among the bottom, middle, and top layered fibers had *p*<0.05. On the other hand, 0.5P had a *p*>0.05, meaning there was no distinct fiber diameter difference in the three layers, implying uniform fiber diameters. Therefore, 0.5P parameters become ideal for PLLA 3D ES.

Suffice it to say that 3D ES presents additional factors compared to traditional 2D ES that are impossible to study even while using DSD. Furthermore, continual in‐depth studies further introduce additional factors increasing complexity. Change in one factor affects the macro and microstructures of electrospun fibers. Despite these challenges, electrospinning remains a fascinating and researchable area that is relevant to different fields due to the diverse materials fabricated. 3D electrospinning expands the possibilities of mimicking naturally occurring matrices and the potential for piezoelectric energy generation. Further research can be geared at closely replicating 3D self‐assembling structures from the designed 3D pattern.

## 4 Experimental Section

### Materials

Poly L‐lactic acid (PLLA) Mw 19000 g mol^−1^ from NatureWorks. N, N‐dimethylformamide (DMF) (99.9% purity) from Sigma–Aldrich, Dichloromethane (DCM) (99.8% purity) from Fisher Scientific, UK, absolute ethanol, Trifluoroacetic acid (TFA) (99.5% purity) from Fluorochem, Phosphoric acid (H_3_PO_4_) (85% in H_2_O) from Acros Organics.

### Experimental Design

A Definitive Screening Design was used to investigate the interaction of process and solution parameters while keeping the ambient conditions constant, as shown in Table 1. The ambient conditions within the 3D ES chamber were kept at 20 ± 1 °C temperature and 50 ± 1% relative humidity and continuously monitored using a digital Temperature/Humidity Meter (TENMARS TM‐183P). Furthermore, the process parameters investigated included working distance (2–6 cm), flow rate (0.2–0.6 mL h^−1^), nozzle speed (1–12 mm s^−1^), voltage (16–20 kV), and collector potential (negative, grounded, and positive). On the other hand, the solution parameters investigated included polymer concentration (9–12 mg mL^−1^) and additive concentration (0–1 wt.%), and the solvents used were DCM/DMF (6:1 v:v) and TFA. Eighteen runs were generated from these eight parameters, as shown in Table 1, generated by Minitab (v 19 2020 2.0).

### Solution Preparation

Weighted PLLA pallets were added to the solvent and mixed using a magnetic stirrer for 24 h. DMF: DCM was based on 1:6 (v:v), while polymer solution: additive concentration was added 10 min before electrospinning. The additive used was phosphoric acid. The conductivity of the polymer solution was measured at ambient conditions by dipping a glass probe conductivity meter (Traceable, Fisher Scientific, Pittsburgh, USA) into the prepared solution.

### Electrospinning Apparatus and Process

The 3D ES apparatus has been described in detail in our previous study.^[^ ^22^ ^]^ It was an enhanced 3D NovaSpider v1.0 (CIC nanoGUNE, Spain) with a nozzle holder, syringe pump, collector plate, and a high‐voltage supplier. The nozzle holder was kept at a required z‐axis while the x‐ and y‐axis moved above the aluminum‐wrapped collector plate. This collector was either positive, grounded, or negatively charged using a DC power supplier (Voltcraft DPPS‐16‐30, Conrad, Germany). The nozzle holder held a blunt 21G hypodermic needle connected to a 30 cm polytetrafluoroethylene (PTFE) tube (2 mm inner diameter) supplying the polymer solution placed in a 5 mL syringe attached to the syringe pump. All experiments ran for 10 min, directed by a G‐code.

### Nozzle Pattern Design

The nozzle pattern was guided using a 55 mm hollow cylinder pattern generated using Onshape (v1.85). Ultimaker Cura (v5.6.0), a slicing software, was then used to generate a G‐code readable by Arduino IDE software that runs 3D ES. This code directs the nozzle pattern movements (*x*‐ and *y*‐axis coordinates (mm)), working distance between the nozzle and collector (z‐coordinates (mm)), extruder movement (mm), and nozzle speed (mm min^−1^). However, the flow rate was calculated using the Equation (1).

(1) $$f_{e}=\frac{\left(\pi r_{e}^{2}\right)*E*F*\;3600\;*10^{-3}}{\Delta X}\left(\mathrm{mL}\;h^{-1}\right)$$

With Flow rate as f_*e*_ (mL h^−1^), syringe inner radius as r_*e*_ (mm), nozzle speed as F (mm min^−1^), extruded distance as E (mm), and ∆X denoting the changes in x‐coordinates (mm). All code edifications were done using Xcode (v. 15).

### Surface and Morphology Characterization

The 3D ES process and 3D macroscopic shapes were captured using an iPhone 12 Pro Max with a 12‐megapixel triple‐lens camera and later converted to jpeg format using Preview (v 11.0). The top and side view pictures were taken next to a 150 mm ruler for later calibration to measure the 3D structural dimensions and height, respectively, shown in Figure 5. (Also see supporting information and Video S1, Supporting Information). The top view pictures also showed the inner diameter (ID), outer diameter (OD), and outermost diameter (OMD). Structures with a height greater than 5 mm were considered 3D.

Sample surfaces were acquired using a Scanning Electron Microscope (SEM) (JOEL JSM‐IT100, JOEL Ltd, Tokyo, Japan). All samples conductivity was increased by 10 nm gold coating for 3 s on a sputter–coater (Desk III, Denton Vacuum, Moorestown, USA). Three sections were observed on 3D structures: top, middle, and bottom layers to show bottom (BF), middle (MF), and top fibers (TF). The material's surface morphology was later analyzed, and ImageJ (v 1.51 w) was used to measure the 80 fiber diameters per sample.

### Statistical Analysis

The presented results are mean ± standard deviation (SD). One‐way analysis of variance (ANOVA) was used for statistical comparisons. A difference was statistically significant when *p* < 0.05.

## Conflict of Interest

The authors declare no conflict of interest.

## Supporting information



Supporting Information

Supplemental Video 1

## Data Availability

The data that support the findings of this study are available in the supplementary material of this article.
