# Supplementary material for: 3D Electrospinning of Macroscopic PLLA Structures
Source: Macromol Rapid Commun. 2025 May 12;46(13):2500130. doi: 10.1002/marc.202500130 (PMC12227234; doi:10.1002/marc.202500130)
Supplement: Supplementary file 1 — Supporting Information [file MARC-46-2500130-s001.pdf]

Macromolecular Rapid Communications

Supporting Information

for *Macromol. Rapid Commun.*, DOI 10.1002/marc.202500130

3D Electrospinning of Macroscopic PLLA Structures

*Yvonne Tusiimire, Michael Lubwama, Robert Tamale Ssekitoleko, Vasileios Koutsos, Wiwat Nuansing and Norbert Radacsi**

Supporting Information

3D electrospinning of macroscopic PLLA structures

Yvonne Tusiimire, Michael Lubwama, Robert Tamale Ssekitoleko, Vasileios Koutsos, Wiwat Nuansing, Norbert Radacsi*

Yvonne Tusiimire, Michael Lubwama

College of Engineering, Design, Art and Technology, Makerere University, Kampala, Uganda

Yvonne Tusiimire

Department of Polymer, Textiles and Industrial Engineering, Faculty of Engineering and Technology, Busitema University, P.o Box 236, Tororo, Uganda

Robert Tamale Ssekitoleko

Department of Physiology, School of Biomedical Sciences, College of Health Sciences, Makerere University, Uganda

Yvonne Tusiimire, Vasileios Koutsos, Norbert Radacsi

School of Engineering, Institute for Materials and Processes, The University of Edinburgh, King's Buildings, Edinburgh EH9 3FB, United Kingdom

Wiwat Nuansing

School of Physics, Institute of Science, Suranaree University of Technology, Nakhon Ratchasima 30000, Thailand; Center of Excellence on Advanced Functional Materials (CoE-AFM), Suranaree University of Technology, Nakhon Ratchasima 30000, Thailand

Norbert Radacsi
School of Engineering, Institute for Bioengineering, The University of Edinburgh, Mayfield Road, Edinburgh, EH9 3JL, United Kingdom
Centre for Cardiovascular Science, The Queen's Medical Research Institute (QMRI), University of Edinburgh, BioQuarter, 47 Little France Crescent, Edinburgh, EH16 4TJ, United Kingdom
E-mail: N.Radacsi@ed.uk

Raw data of samples

a) SEM images for DSD runs (1-18)

b) Raw data for 0F, 0G, 0P, 0.5F, 0.5G, 0.5P, 1F, 1G and 1P showing SEM images, top and side views

Results include

a) Fiber diameter measurements for DSD runs

b) Fiber diameter measurements for 0F, 0G, 0P, 0.5F, 0.5G, 0.5P, 1F, 1G and 1P from bottom, middle and top layers

Raw data samples

a) SEM images for DSD runs (1-18)

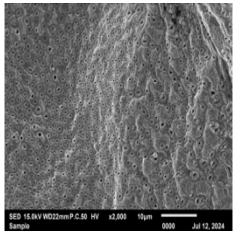

DSD 1

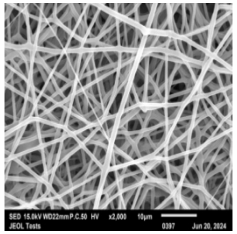

DSD 2

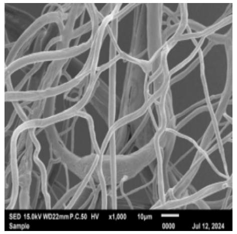

DSD 3

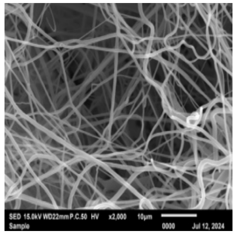

DSD 4

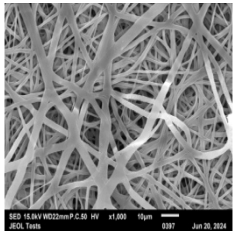

DSD 5

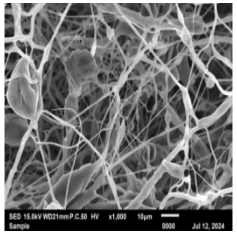

DSD 6

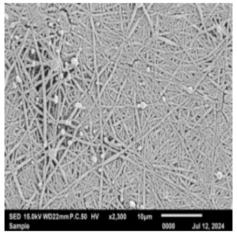

N/A

N/A

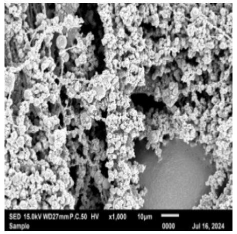

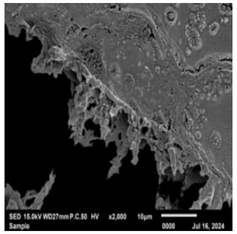

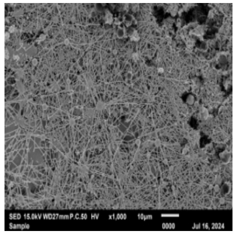

DSD 7	DSD 8	DSD 9	DSD 10	DSD 11	DSD 12
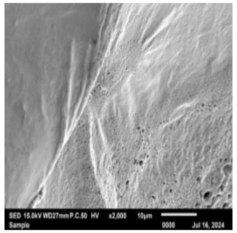	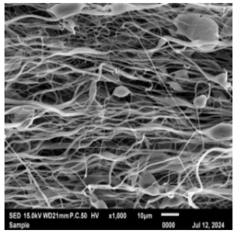	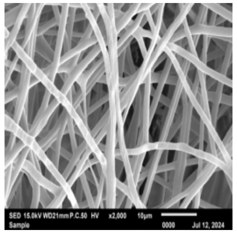	N/A	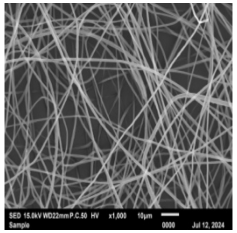	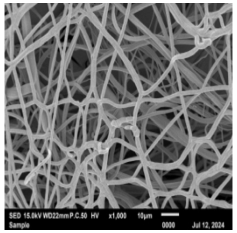
DSD 13	DSD 14	DSD 15	DSD 16	DSD 17	DSD 18

b) Raw data for 0F, 0G, 0P, 0.5F, 0.5G, 0.5P, 1F, 1G and 1P showing SEM images, top and side views

	0F	0G	0P	0.5F	0.5G	0.5P	1F	1G	1P
BOTTOM	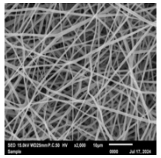	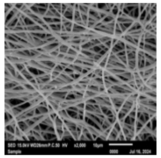	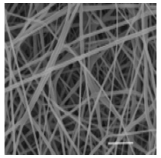	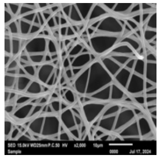	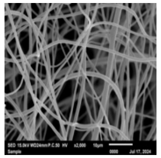	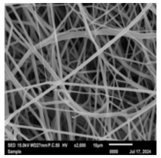	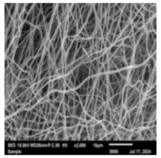	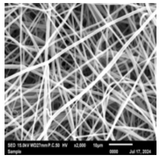	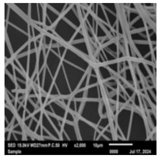
MIDDLE	N/A	N/A	N/A	N/A	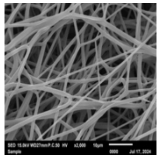	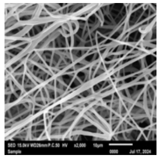	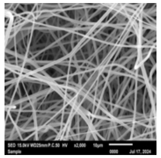	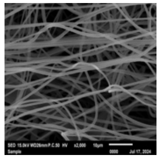	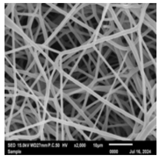
TOP	N/A	N/A	N/A	N/A	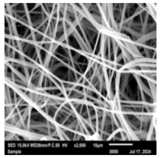	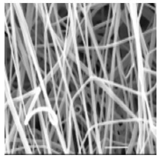	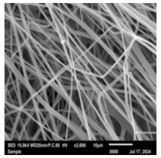	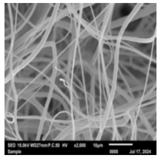	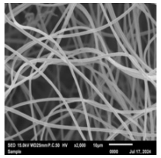

TOP VIEW	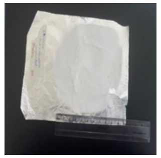	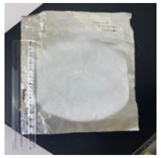	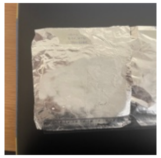	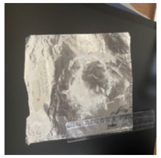	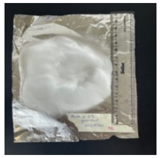	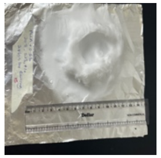	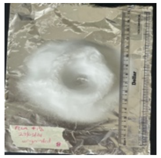	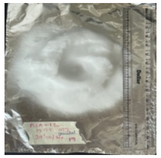	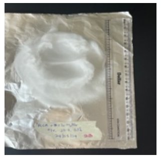
SIDE VIEW	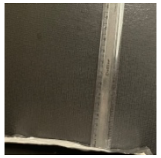	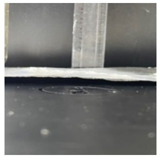	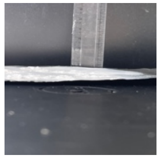	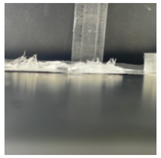	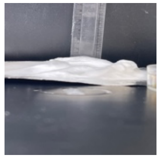	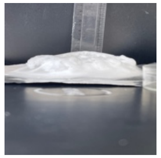	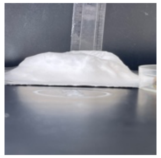	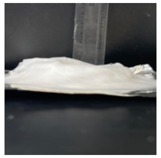	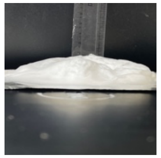

Results include

a) **Fiber diameter measurements for DSD runs**

DSD 2	DSD 3	DSD 4	DSD 5	DSD 6	DSD 14	DSD 15	DSD 17	DSD 18
0.3041	3.8886	0.7142	1.3687111	2.0272	0.5044	1.4901	1.0177	3.0973
0.6002	1.3372	0.583	1.6044208	0.5398	0.6686	1.2769	0.9847	1.7532
0.8645	1.773	0.5206	1.7081258	0.4678	0.5252	1.5934	0.9452	1.2985
0.8222	1.6002	2.1439	2.8553551	0.878	0.1501	1.2232	1.0889	3.8267
1.2216	4.6729	1.087	2.1508534	0.411	0.1678	1.3543	1.2446	2.0126
0.5758	3.712	0.6823	0.8233434	0.8984	0.6411	1.4329	0.8164	1.7934
0.5814	1.7441	0.7284	1.6355226	1.054	1.8562	1.3606	1.1899	2.2228
1.3	1.3698	0.4072	1.2715618	5.1243	2.3141	1.1772	1.0407	2.3994
0.5544	2.1561	0.6393	2.0314799	0.7476	1.3155	1.2186	1.1096	1.6728
0.5303	8.2616	0.6082	1.8285699	1.5133	0.8164	1.3272	0.8918	2.4932
0.596	1.0121	0.6071	1.0700896	0.9648	0.4477	1.4559	1.2178	2.6072
1.0354	0.8663	0.4903	1.4858455	0.4785	0.8335	1.5052	0.86	1.8748
1.1408	2.3548	0.9024	1.9154305	1.795	0.2705	1.023	0.9769	2.6248
0.7828	1.4512	0.4857	1.5972678	1.9589	0.4514	1.4166	1.0681	1.1627
0.6082	2.8947	0.6738	1.0174296	0.4726	0.3752	1.5141	0.9977	1.0714

1.0164	1.7147	0.6211	1.2929942	7.8834	0.9731	1.7028	1.0645	2.3673
0.6823	1.4588	0.505	1.7764776	4.2357	0.2443	1.8451	1.0383	3.6816
0.6643	2.1612	1.0214	2.165709	4.1229	0.3322	1.5719	1.0815	2.0384
0.8967	1.9406	0.3788	1.4843034	0.5324	1.0205	1.5208	0.962	1.3112
1.0549	2.3027	0.441	2.3841634	0.7103	0.6618	2.1285	0.8567	1.7913
0.7889	2.4431	1.1114	0.7444625	1.5882	1.1478	1.6184	1.053	2.4685
1.135	1.9933	0.7284	1.1505329	0.411	0.4674	1.3072	1.3942	1.7503
0.9192	2.5527	0.7354	1.2424104	0.8137	0.6711	1.2531	1.0708	1.4956
0.7796	1.4858	0.5607	1.2200898	1.2196	0.6446	1.652	1.4254	2.3823
0.9035	3.7262	0.7187	1.3603192	0.9648	0.6454	1.1897	1.0889	1.8856
0.9024	2.9633	0.3517	1.722811	2.2441	0.6844	1.678	1.0886	1.3218
0.8957	2.0339	0.583	1.3535681	0.7392	0.5744	1.6599	1.1245	2.2817
0.6061	3.7926	0.8085	1.1584677	2.0965	0.5369	1.2435	1.0333	1.5393
0.8689	6.5784	0.3846	1.5133352	1.3842	0.6384	1.5308	1.1208	1.4785
0.7021	1.1604	0.5357	3.3621935	2.7347	0.7718	1.2593	0.8427	2.1433
0.7035	1.7237	1.2438	1.8285699	0.9245	0.5044	1.8943	1.1448	1.9232
0.7796	2.6908	0.5327	1.5972678	5.0952	0.8627	2.0284	1.3757	1.8048
0.7362	2.844	1.0797	0.9618909	1.1527	0.5379	2.0298	1.4718	1.8911
0.5107	1.6611	0.8795	1.9058413	0.5763	0.9085	1.3636	1.0921	1.9922
0.8349	3.7926	0.7746	1.7596386	1.5166	1.1255	1.5212	0.8622	0.9105
0.859	1.9131	0.6358	1.4388627	0.6468	0.522	1.6509	1.0466	0.6585
0.8272	2.7748	0.3993	1.0912815	1.3854	1.0743	1.2186	0.786	1.8245
1.0621	3.1762	0.4697	3.5465035	2.742	1.132	1.6182	0.889	1.6544
0.8021	3.2094	0.2945	2.0483203	0.7135	1.807	1.4698	0.7712	1.3868
0.9715	1.8075	0.2036	1.3886448	1.1468	0.6644	1.2116	1.0824	2.0259
0.8755	4.097	0.3234	2.1794129	0.7476	0.5122	1.6461	0.8351	1.0808
0.8638	4.5543	0.7508	1.6466867	1.3743	0.4839	0.6944	1.0648	1.2856
0.8882	3.3209	0.5944	2.240554	2.2017	0.7413	1.7248	1.1648	2.1097

0.7909	1.7592	0.4048	1.2715618	1.2865	7.3993	1.4698	1.1073	1.7201
0.7323	2.063	0.4974	0.8149559	1.9808	5.0672	1.2041	0.9288	1.8687
0.8935	1.833	0.9702	0.5154234	1.857	0.8164	1.8246	0.8744	2.2365
0.7393	12.1449	0.6328	1.0330661	0.6538	0.4271	1.2543	0.8918	1.233
0.9265	3.4166	0.5463	1.0700896	1.1293	0.7181	1.5618	1.1593	1.4471
0.8867	1.4588	0.6388	1.460976	1.3367	1.1078	1.3963	0.9553	1.3969
0.9435	2.4692	0.5422	2.3149577	1.6218	0.9159	1.3499	0.9128	1.9331
0.836	3.2585	0.6667	1.3552591	1.1956	1.1488	1.4979	0.8567	2.1927
0.7779	7.2498	0.3051	1.4858455	3.1329	0.3039	1.7177	0.9075	1.5151
0.8645	1.8245	0.5538	1.3803741	3.7039	2.3114	1.4203	0.866	1.751
1.0226	1.4017	0.9192	1.264337	4.7626	0.9214	1.5808	0.9145	1.8293
0.9313	2.3065	0.3082	3.2801344	0.5005	0.5078	1.6114	0.7107	2.2274
0.6785	2.9304	0.4798	1.2876695	2.0935	0.7783	1.1812	0.7712	2.1575
0.8245	1.5682	0.5544	1.6742713	1.214	0.6077	0.8854	1.1423	1.372
1.0282	3.6015	0.5181	1.2588911	1.9532	5.2742	1.8122	0.8611	1.6169
0.6566	1.5166	0.544	2.1825631	2.7751	0.6885	1.2791	1.302	2.2359
0.7111	1.0506	0.6628	1.1263932	5.2965	0.9937	1.1421	0.9986	1.998
0.9718	4.6326	0.404	1.733413	4.073	0.745	1.6828	1.1689	2.0328
0.7427	1.1432	0.505	1.2257081	1.9575	0.5209	1.4901	0.937	2.5398
0.7142	1.5297	0.7151	1.1973533	2.4761	1.4433	1.613	1.2384	1.1349
1.0439	2.0694	0.6082	1.4904623	3.6379	1.0451	1.1878	1.249	1.1517
0.9384	3.0909	0.5273	1.9142344	1.5811	0.7652	1.5349	0.9928	1.8931
0.8152	2.2007	0.6165	1.0241601	1.2887	2.8014	1.8853	1.2779	2.3956
0.7597	3.2142	0.43	1.0571705	1.6632	1.7449	1.2673	1.2218	1.8293
0.5764	0.8939	0.589	1.3018202	0.4285	0.2149	1.4132	1.0578	1.9429
0.9529	2.4719	0.4142	1.7400065	0.5734	0.242	1.581	1.167	1.5947
0.844	1.7592	0.6609	1.9812558	2.6744	0.3614	1.7892	0.8491	2.1049
1.1871	1.725	1.0861	1.3000598	2.6448	1.1255	1.5376	1.0756	0.7905

0.9848	2.5432	0.6934	1.453117	3.1413	0.4055	1.002	0.8652	0.9961
0.7597	1.5961	0.6071	1.9154305	1.4384	0.6169	1.3909	1.3032	0.5369
0.7362	1.2781	0.4903	1.2442524	0.6734	15.0672	1.4798	0.873	0.7985
0.8085	6.0498	0.9091	1.3703833	0.8857	6.1306	2.1507	0.7729	2.6772
1.0255	1.8462	0.6738	1.7557297	0.7566	0.7503	2.3019	0.9215	1.9928
0.8967	1.5497	0.8406	1.2588911	2.3506	0.7725	1.6476	1.1457	1.0654
0.8307	2.5639	1.2494	3.0243995	1.1571	0.5714	1.6934	1.2934	1.2876
1.0192	2.3772	0.9105	1.520883	1.8577	0.7747	1.3291	1.0815	0.9216
1.0412	1.3682	0.7508	1.7254676	1.078	0.9085	1.4587	1.1336	0.8159

b) Fiber diameter measurements for 0F, 0G, 0P, 0.5F, 0.5G, 0.5P, 1F, 1G and 1P from bottom, middle and top layers

0F			0G			0P			0.5F			0.5G			0.5P			1F			1G			1P		
Bottom	Middle	Top	Bottom	Middle	Top	Bottom	Middle	Top	Bottom	Middle	Top	Bottom	Middle	Top	Bottom	Middle	Top	Bottom	Middle	Top	Bottom	Middle	Top	Bottom	Middle	Top
0.7812626	0	0	0.7356716	0	0	0.547375	0	0	0.9600459	0	0	1.3550079	0.977196	1.1557177	0.4780462	0.805761	0.8394302	0.5009702	0.8544884	0.4020101	0.6642881	0.6113329	0.5743957	1.0483709	2.284289	0.971484
0.6998712	0	0	0.5896574	0	0	0.5978357	0	0	0.977316	0	0	0.7347215	1.1821419	0.9886392	0.7325293	0.5958767	1.1024652	0.5009702	0.7111153	0.5618261	0.6358403	0.9323606	0.6236483	0.7423739	1.1658475	1.3131313
0.6879849	0	0	0.9081993	0	0	0.6443569	0	0	0.9380953	0	0	0.9528498	0.7006917	0.7527123	0.5887104	0.6086469	0.6685939	0.5407621	0.41108	0.7419509	0.7129065	0.8475944	0.766091	0.8968096	0.6054662	1.2047334
0.7459493	0	0	0.7408283	0	0	0.783381	0	0	1.0850631	0	0	2.1293197	0.5421307	0.6404613	0.5179765	0.7445769	0.8870764	0.3773658	0.6313111	0.7658166	0.6081594	0.9577447	0.8285584	0.8153231	0.7419218	1.0226493
0.9485224	0	0	0.8384543	0	0	0.717683	0	0	0.8922224	0	0	1.1395066	0.9609276	0.7341772	0.8230295	0.7169497	0.6438135	0.4407847	0.9312641	0.7115479	0.7111153	0.7355418	0.7128953	0.7495672	0.8199198	1.2298783
0.8844861	0	0	0.540829	0	0	0.5074785	0	0	0.9265845	0	0	1.1181312	0.8594798	1.5109492	1.087678	0.737224	0.7729888	0.3617228	0.7111153	0.8501972	0.783233	0.98504	0.5575804	0.8890327	1.7204028	1.191962
0.647605	0	0	0.8262579	0	0	0.6270655	0	0	1.0659868	0	0	1.3467	0.8449449	0.4668124	0.5056419	0.8292803	0.7070685	0.489583	0.8784077	0.9326991	0.6328245	0.5784354	1.6496337	1.4620393	1.6211612	1.3794445
0.8339015	0	0	0.8597505	0	0	1.5459772	0	0	1.2319208	0	0	0.8151003	0.7428169	1.1931009	0.689924	0.6081202	0.7929428	0.3542394	0.6555916	0.5329951	0.5606953	0.5844072	0.9826906	0.9551014	1.1902633	1.3738859
0.597142	0	0	0.7124502	0	0	0.6310596	0	0	0.9048687	0	0	0.7820751	1.3819638	0.7306769	1.1776889	0.5926411	1.1182585	0.4938839	0.8143669	1.2372937	0.3676811	0.696755	0.9224097	0.8352119	0.8011325	1.4444798
0.7097734	0	0	0.8262579	0	0	0.372582	0	0	1.0603458	0	0	1.4106022	0.5305835	0.7852184	1.0498752	1.3032401	1.6760092	0.504895	0.6822835	1.0567709	0.5544048	0.8091076	0.8123182	0.7825308	0.70128	1.2142238
0.6055826	0	0	0.7677456	0	0	0.6990617	0	0	0.9567524	0	0	1.1614551	0.8935253	0.9873418	0.689924	0.7359187	0.582998	0.4677865	0.7642777	0.5324025	0.3787867	0.7792996	1.1435324	0.8586911	0.6800489	1.1627136
0.5094143	0	0	0.684286	0	0	1.2808914	0	0	1.2041929	0	0	1.0519344	1.0664596	0.7341772	0.6522972	1.1213754	1.0399593	0.4561892	0.8956612	0.5956417	0.8439758	0.7885605	0.6626169	0.9695337	1.0752518	1.2047334
0.6782323	0	0	0.8123182	0	0	0.7181205	0	0	0.7933469	0	0	1.172993	1.0189414	0.7599155	0.9062629	0.8510219	0.8367671	0.4938839	0.6934084	0.6420569	0.7840467	1.0175104	1.5167828	1.0815666	0.8866753	1.0214014
1.0125819	0	0	0.6500498	0	0	0.6559504	0	0	1.2811566	0	0	0.7122286	1.2196498	0.8857142	0.7740137	0.8857114	0.8518724	0.4037205	0.8585831	0.989835	0.6002438	0.879397	0.8123182	1.4213157	0.7365814	1.1213941

0.7217405	0	0	0.4644607	0	0	0.7510415	0	0	1.1392712	0	0	0.763927	0.7725412	0.6625951	0.8091051	0.6128445	0.9192029	0.4433363	0.7182534	0.730374	0.8488729	0.696755	0.4192271	0.9669257	0.7862099	1.2298783
0.7953478	0	0	0.5901952	0	0	0.6785436	0	0	0.9214604	0	0	0.7770363	0.6719793	0.5063291	0.4878499	1.2178202	0.7973537	0.5941814	0.6709741	0.910089	0.7905266	0.6281407	1.1085985	0.900673	0.6915621	0.9609241
0.5037783	0	0	0.7265595	0	0	0.5111783	0	0	1.0733639	0	0	1.1468695	0.6454827	0.5212977	0.7805113	1.0743811	0.9625787	0.7222752	0.5538294	0.5865637	0.8025352	0.6765282	0.6654834	0.7419486	0.7599468	1.3551927
0.8224095	0	0	0.7689843	0	0	0.6771536	0	0	1.0181274	0	0	0.9020263	1.5219517	0.7611796	0.3908618	0.5958767	0.6822835	0.4808657	0.8131915	0.6435301	0.8809448	0.9132051	0.5044077	1.1883614	1.5558334	1.0411883
0.4961641	0	0	0.3853163	0	0	0.6285663	0	0	1.1194271	0	0	2.4572046	1.1119491	0.7093127	0.66095	0.9127949	0.36156	0.5098968	0.7893156	0.8352146	0.5050489	0.7641661	0.4812336	1.0301475	1.0081163	0.8834776
0.6066294	0	0	0.5743957	0	0	0.753963	0	0	0.9059145	0	0	1.1458905	0.6686194	0.5844758	0.6082251	0.7411258	0.7579941	0.468389	0.7642777	0.4473491	0.5050489	0.7495695	0.5044077	0.8586911	0.9136168	1.7378435
0.7408283	0	0	0.681964	0	0	0.5726127	0	0	0.9567524	0	0	1.2467376	1.0117324	0.5931835	1.1625828	0.6816642	0.9066296	0.3916664	0.7178093	0.5300257	0.7634428	0.7641661	0.4596546	1.0498752	0.9427821	0.7575758
0.5987337	0	0	0.7093263	0	0	0.8480637	0	0	0.9698592	0	0	0.964556	0.6123586	0.7913164	0.7247316	0.6914662	0.6565636	0.4333651	0.7151392	0.8883251	0.8722152	0.9238581	0.6087177	0.9534475	0.9337658	0.8159341
0.8912749	0	0	0.7885378	0	0	1.125586	0	0	0.8184144	0	0	0.8901281	0.7110611	0.7093127	0.6301456	0.7341749	0.9104898	0.6054643	0.7909298	0.7093263	0.5205925	0.6077079	0.6568466	0.7756432	0.866733	1.1659996
0.766091	0	0	0.6930386	0	0	0.7285412	0	0	0.813773	0	0	0.7192844	0.9281421	0.6793118	0.5865618	0.8415553	0.8637665	0.4656719	0.7579941	0.7541875	0.5181368	0.8989218	0.6568466	0.899621	0.6192809	1.4356233
0.6553961	0	0	0.7217405	0	0	0.6535521	0	0	1.0140891	0	0	0.7098611	0.8555402	0.9873418	0.6633335	1.0333299	0.8949489	0.5230012	0.7579941	1.1747407	0.6081594	1.2562814	0.3811775	1.2431435	1.0497733	0.7088722
0.6875236	0	0	0.879811	0	0	0.4607699	0	0	1.001246	0	0	1.0188753	0.5773373	1.0874294	0.6862541	0.94522	0.8867169	0.4808657	0.7426967	0.6159623	0.7186972	0.5956417	0.762355	0.8546381	1.1639109	0.8405716
0.8597505	0	0	0.5295667	0	0	0.6734329	0	0	1.1058096	0	0	0.6700083	0.8287979	1.1689515	0.9380953	0.6349314	0.4642605	0.2869514	0.8870764	0.6788571	0.6565636	0.8295263	0.462407	0.7194862	1.3932808	1.0654052
0.5044077	0	0	0.6875236	0	0	0.8331187	0	0	0.8427365	0	0	0.9949116	0.7976419	0.6252703	0.8295237	0.4986747	0.9104898	0.5559326	0.6438135	0.9498019	0.7840467	0.7654043	1.1074533	0.8851186	0.6165427	0.7016386
0.5944798	0	0	0.7343768	0	0	0.9596055	0	0	0.977316	0	0	1.2507818	0.732897	0.7884765	0.6595157	0.5340495	0.7596748	0.4583476	1.3778211	0.9296482	0.6333281	0.8996238	0.7489203	0.530024	0.7731899	1.1122584
0.4838633	0	0	0.6805671	0	0	0.8771902	0	0	0.8904517	0	0	1.0683429	0.9419912	0.6409615	0.6676024	0.6409594	0.8394302	0.4992786	0.631816	2.8541652	0.6272577	0.8397374	0.9465136	1.232177	0.7124502	0.9104927
0.7160035	0	0	0.6175139	0	0	0.9848031	0	0	0.981184	0	0	0.9198899	0.8109427	0.7380952	0.66095	0.9623553	1.1419579	0.4656719	0.7575733	0.6459779	0.68972	0.9310054	1.1586902	0.9350621	0.9762849	0.8585859
0.6539423	0	0	0.766091	0	0	0.6061829	0	0	1.0989376	0	0	0.8157884	0.8389262	0.8105221	0.502511	0.6128445	0.8838356	0.4656719	0.4980561	0.628643	0.9822519	0.683491	1.4713433	1.2477055	0.7833353	0.9104927
0.5815313	0	0	0.7003244	0	0	0.8342489	0	0	0.8241793	0	0	0.5232932	1.3422819	0.8491397	0.6537473	0.6587124	0.825643	0.7496706	0.7596748	0.7691069	0.5606953	1.0313757	1.4092293	0.6296444	1.2575412	1.015139
0.5815313	0	0	0.7304786	0	0	0.6578628	0	0	0.8851186	0	0	0.7347215	0.8116367	0.917001	0.7740137	0.8540291	0.6081594	0.3952499	0.7002717	0.7740162	0.915031	0.5276382	1.0153977	0.7247316	1.1423914	1.3476428
0.6342407	0	0	0.7343768	0	0	0.9596055	0	0	0.8352119	0	0	0.6039373	1.0408096	1.001521	0.7691045	0.6914662	0.6785346	0.4433363	0.4903138	0.8241819	0.8439758	0.8180312	0.8567935	1.2128121	1.2744696	0.7283942
0.5192828	0	0	0.5815313	0	0	0.9327186	0	0	0.7541851	0	0	0.6231437	0.65156	0.8117073	0.6532643	0.5604018	0.9178143	0.4446066	0.5350897	0.7461931	0.825643	0.5972294	1.6808766	0.8367223	0.8342396	1.2656529
0.4792518	0	0	0.5337436	0	0	1.2290889	0	0	0.9238552	0	0	0.9045112	0.7570815	0.6572534	0.6205553	0.5910167	0.8928087	0.3698177	0.7456958	1.0040825	0.6452976	0.8427392	1.8153504	0.7756432	1.1047445	0.9104927
0.696691	0	0	0.7322137	0	0	0.6285663	0	0	0.7393916	0	0	1.4213022	0.7259499	0.8806343	1.0040793	0.9046841	0.7235608	0.17289	0.6869408	1.0329048	0.6628466	0.8352146	1.2132589	1.129253	0.8123182	1.1714559
0.647605	0	0	0.540829	0	0	0.8480637	0	0	0.8446072	0	0	0.8157884	0.9305654	0.917001	0.731667	0.6910025	0.8025352	0.2496393	0.6609197	0.8634593	1.0179586	0.8214966	0.7322137	0.7106578	1.4249003	1.0411883
0.7759658	0	0	0.5928767	0	0	0.856539	0	0	0.8044101	0	0	0.9045112	1.1399522	0.829283	0.6484144	0.5163548	0.8619189	0.7422994	0.7929428	0.8367249	0.7680231	0.7234261	0.7252484	0.8575877	1.3538636	1.103047
0.3606504	0	0	0.3115697	0	0	0.7216108	0	0	1.0689437	0	0	0.9265757	0.8489336	0.7699699	0.6133928	0.5364444	0.354435	0.5963132	0.8025352	0.7039661	0.5272865	0.6077079	1.3463706	0.7760501	0.8088393	1.5335582

0.7706327	0	0	0.701682	0	0	0.8182845	0	0	0.7756432	0	0	0.9439749	1.009504	1.0991541	0.5179765	0.8172132	1.1433531	0.2479391	0.542193	1.0359562	0.8765909	0.5784354	0.9810752	0.6999168	0.8171637	1.0902542
0.5632413	0	0	0.8138788	0	0	1.0075032	0	0	0.813773	0	0	1.2756578	0.5860486	1.3032443	1.0230758	0.7380928	0.7929428	0.4171185	0.6666837	0.9296482	0.7680231	1.0733673	0.9591654	0.9323576	1.1682638	1.4681254
0.6568466	0	0	0.7164465	0	0	1.1498781	0	0	0.7654019	0	0	0.9960389	0.8287979	0.7160575	1.184104	0.7306746	0.6984481	0.5642399	1.0575922	0.4322777	0.5081956	0.8075457	0.9130765	0.7234238	1.2132589	1.4956659
0.5875015	0	0	0.4864788	0	0	1.1053137	0	0	0.9407832	0	0	1.0433645	0.9159291	0.7594937	0.6405783	0.5711634	0.6609197	0.4044184	1.096375	0.853532	0.8333307	0.7325316	0.7706327	0.6361281	0.8738614	1.0925913
0.5632413	0	0	0.8388325	0	0	0.497478	0	0	0.9567524	0	0	0.901404	0.7666886	0.4668124	0.66095	0.5910167	0.9744303	0.4677865	0.8337132	0.7186105	0.6822835	0.6857962	1.2383626	1.2372898	1.203926	1.050942
0.8339015	0	0	0.4705678	0	0	1.6588701	0	0	0.9020737	0	0	1.2286023	0.9257124	0.4869717	0.7495672	0.5552314	0.7579941	0.5778202	0.8707518	1.136223	0.7461232	0.8363476	0.7128953	0.6484144	0.8757955	1.5193544
0.6805671	0	0	0.691664	0	0	0.8055192	0	0	0.9200892	0	0	0.6832771	0.9281421	0.8860759	0.5784336	0.6180515	0.7779229	0.4881409	0.5254693	0.5300257	0.8585831	0.7111041	1.1222503	0.991425	0.631009	1.3645713
0.5241474	0	0	0.8990715	0	0	0.8554383	0	0	0.6281387	0	0	0.964556	0.7570815	0.6454455	0.6765261	0.6741005	0.9125886	0.5896556	0.7270775	0.7247339	0.6328245	0.7558597	0.7124502	1.3058036	0.7753751	1.1213941
0.4845185	0	0	0.7322137	0	0	0.8775482	0	0	0.9407832	0	0	0.6435219	0.7006917	0.8105221	0.8386064	0.6816642	0.6642881	0.6233636	0.5368743	0.5533346	0.7696819	0.5025126	0.6302265	0.9695337	0.8503099	0.9312671
0.5592847	0	0	0.6436742	0	0	0.9876691	0	0	1.0162656	0	0	0.569507	0.8724832	0.6123234	0.7604371	0.6469312	1.1717243	0.3832973	1.0609033	0.7654043	0.8194409	0.9955584	1.5649597	1.2494751	1.117939	1.1516923
0.4289518	0	0	0.6930386	0	0	1.1095676	0	0	1.1205544	0	0	0.9755468	0.944975	0.6910048	0.7558574	0.6840108	0.8084727	0.5037767	0.6081594	1.0839022	0.5012467	0.8352146	0.8608568	0.8295237	0.9617344	0.7456981
0.5660505	0	0	0.5682879	0	0	0.7268148	0	0	1.1038097	0	0	1.3103759	0.8489336	0.7766006	0.8397348	0.6489096	0.7111153	0.3728553	1.6799994	0.5972294	0.5813549	0.3490564	1.0582343	0.7146441	0.7116581	1.4293911
0.7594364	0	0	0.7514576	0	0	0.8480637	0	0	0.9471365	0	0	1.1527259	1.0595741	0.8788129	0.9104329	0.7949503	0.8306481	0.597966	0.6628466	0.8367249	0.41108	0.7885605	0.9585037	1.0975005	0.987771	0.798555
0.6852126	0	0	0.4192271	0	0	0.6056646	0	0	0.9827912	0	0	1.1261323	0.9609276	0.9516428	0.8586911	1.0539034	0.8131915	0.6835623	0.8589544	0.7199271	1.0390391	0.8542714	1.0936168	0.8184144	0.9687459	0.9394482
0.5192828	0	0	0.7373945	0	0	0.6968118	0	0	1.0347334	0	0	0.8735839	0.9783476	1.7730558	0.6030132	0.5931816	0.9104898	0.4521535	0.6023648	0.8295263	0.8394302	0.7039661	0.7845044	0.8707371	0.8067447	0.7575758
0.8130989	0	0	0.540829	0	0	0.8285826	0	0	1.1376076	0	0	1.076714	1.0925383	1.1336007	1.0202953	0.6683715	0.9216277	0.5731652	0.582998	0.6395941	0.6551051	0.5423878	1.0385656	0.7008182	0.9917597	0.9312671
0.5069172	0	0	0.8316158	0	0	0.8421175	0	0	0.8102746	0	0	0.9020263	1.2537947	0.6567657	1.7732658	0.7514316	0.8967285	0.5523705	1.065701	0.9214634	0.7016363	1.1574164	0.9455075	1.1560483	1.0236608	0.9312671
0.5944798	0	0	0.6256797	0	0	0.5604163	0	0	0.981184	0	0	0.8273992	0.9305654	0.6567657	0.7825308	1.2973253	0.7905266	0.6303364	0.5959791	1.1392748	0.7634428	1.0659901	1.4081033	0.9630004	1.2859237	1.0654052
0.7780073	0	0	0.7252484	0	0	1.1366922	0	0	0.8883223	0	0	0.9344154	1.2609593	0.4869717	0.6220794	0.8729562	0.3796275	0.527297	0.7288295	0.5729525	0.582998	0.6551962	1.3000995	0.6071863	0.7614297	1.0226493
0.681964	0	0	0.6256797	0	0	0.9940085	0	0	0.607706	0	0	1.1701192	1.7517293	0.5689166	0.6296444	0.5844739	0.6799429	0.451217	0.7075192	0.6760615	0.6869408	0.5844072	0.8912749	0.7817236	1.030251	0.6738214
0.8608568	0	0	0.6421939	0	0	0.6325509	0	0	0.6981106	0	0	1.1136054	1.0067114	0.8950719	0.6834888	0.6910025	0.6555916	0.5963132	0.888154	0.5371246	0.8895888	0.7106601	0.9591654	0.7933469	1.1600279	1.0618079
0.4192271	0	0	0.4890803	0	0	0.8274447	0	0	1.0329015	0	0	1.0055706	1.1038177	0.6117998	0.6537473	0.8105195	0.8841962	0.4890067	1.0056689	0.9773191	0.8084727	1.156052	1.01914	1.2314082	1.2317126	0.6102548
0.5100367	0	0	0.5100367	0	0	0.7375385	0	0	0.7825308	0	0	1.0422883	0.5122261	0.6645268	0.6834888	0.8089364	0.7288295	0.456807	0.621128	0.9245412	0.6984481	0.9669287	1.0912937	0.8904517	0.7983116	1.1122584
0.4561907	0	0	0.7459493	0	0	0.7306934	0	0	0.9299848	0	0	0.9100775	0.944975	0.6334175	0.7558574	0.7249506	0.8084727	0.5604791	0.6144185	0.4632937	0.9192029	0.8586938	0.8111457	0.8016586	0.8342396	1.0843894
0.4812336	0	0	0.659257	0	0	0.4600878	0	0	0.7303717	0	0	0.8939026	1.0451283	0.6533412	0.7885581	0.9963851	0.7709237	0.5216516	0.6358403	0.5887123	0.5959791	0.6077079	1.0413112	1.2010433	0.9791691	0.9529274
0.9505271	0	0	0.7476485	0	0	0.7518773	0	0	0.8775977	0	0	0.7169402	0.8808325	0.7341772	0.6341402	0.626804	0.5986481	0.527297	0.621128	0.9551044	0.7340604	0.8797558	1.1589639	0.9214604	1.0214547	0.7905291
0.7594364	0	0	0.8597505	0	0	1.3302068	0	0	0.813773	0	0	1.0038951	1.6524929	0.7346136	0.7008182	0.6659698	0.8337132	0.4417432	1.2689196	0.9827943	0.6236894	0.9310054	1.3350126	0.8321831	0.9511202	0.8718523
0.540829	0	0	0.7128953	0	0	0.8984156	0	0	0.9620165	0	0	0.6906287	1.1873695	0.8875214	0.6551942	0.8385034	1.314098	0.5173089	0.7088699	0.9296482	0.5769507	0.8352146	0.8251053	0.7889582	0.8260872	1.0701828

0.5743957	0	0	0.604534	0	0	0.7375385	0	0	0.8230295	0	0	0.8102669	0.9960283	0.8265736	0.685794	0.5739619	0.511323	0.4728827	0.5873569	0.5025126	0.6822835	0.8475944	0.462407	1.176348	0.987771	1.2142238
0.5880412	0	0	0.5337436	0	0	0.6097988	0	0	1.0359529	0	0	1.4086118	0.9401964	0.7380952	0.6241057	0.7745321	1.0787849	0.6394416	0.6492383	0.5050189	0.7327562	0.628643	0.8123182	0.907655	0.6274237	0.7508116
0.5343376	0	0	0.8095798	0	0	0.7306934	0	0	1.0838988	0	0	0.7791998	1.1648695	0.7749482	0.6281387	0.7909088	1.0701794	0.5365741	1.0551776	0.6633356	0.4552449	0.9218058	1.2208182	0.8851186	1.0197969	0.959596
0.6626169	0	0	0.8173794	0	0	0.9930602	0	0	1.2041929	0	0	1.1844185	1.5333771	0.6508841	0.6765261	0.7038681	0.6822835	0.5208401	0.8131915	0.628643	0.5895243	0.8180312	0.9481879	0.8423619	1.2165085	1.2131729
0.5295667	0	0	0.7356716	0	0	0.6025452	0	0	1.1483778	0	0	0.540178	0.5773373	0.8246328	0.7541851	0.8381211	1.2134319	0.4546413	0.5895243	1.7987212	0.4903138	0.9214634	0.5547284	0.7658142	1.6456759	1.0925913
1.052523	0	0	0.7097734	0	0	0.6265644	0	0	0.9853573	0	0	0.7316601	0.7078867	0.6601724	0.5618244	1.0539034	1.0282428	0.423825	0.7392543	0.8501972	0.6666837	0.838609	0.5564414	0.8321831	0.9409858	0.6775964
0.5987337	0	0	0.7057393	0	0	0.7306934	0	0	0.8055864	0	0	2.3017979	1.0664596	0.6645268	0.6205553	0.7341749	0.7667767	0.5270296	0.8409481	0.8879697	0.7235608	0.9048715	1.3003435	0.900673	0.9409858	1.0654052
0.4999857	0	0	0.5842526	0	0	0.753963	0	0	1.1092297	0	0	0.9045112	0.7359635	0.7552625	0.8118313	0.7302359	0.8484972	0.4938839	0.6551051	0.70531	0.5205925	0.6341422	1.5354912	0.9107795	1.3720704	1.0925913
0.7003244	0	0	0.7252484	0	0	0.6621455	0	0	0.7853493	0	0	0.5704915	0.7412994	0.577304	0.8184144	0.6017634	0.7864829	0.6213246	1.3598858	0.5784354	1.1156895	0.7541875	0.4169508	0.8721859	0.8790094	1.1822929
0.5069172	0	0	0.6436742	0	0	0.9474185	0	0	0.9214604	0	0	0.9457565	0.9937646	0.8918438	0.4832996	0.724066	0.8678174	0.527297	0.5462939	0.5740532	0.7840467	0.5179781	0.7476485	1.0252332	0.866733	1.1213941
0.5842526	0	0	0.5875015	0	0	0.9977928	0	0	0.9132022	0	0	0.9668802	0.839597	1.285418	0.9850369	0.7088585	1.0301017	0.5208401	0.6585032	1.2474564	0.7533528	0.6857962	0.3535433	0.8230295	0.8308525	1.115693
